# Women (Do Not) Belong Here: Gender-Work Identity Conflict among Female Police Officers

**DOI:** 10.3389/fpsyg.2017.00130

**Published:** 2017-02-06

**Authors:** Jenny Veldman, Loes Meeussen, Colette Van Laar, Karen Phalet

**Affiliations:** Center for Social and Cultural Psychology, Department of Psychology, KU Leuven, Leuven Belgium

**Keywords:** conflicting identities, gender diversity, team identification, support, diversity climate, well-being, work motivation

## Abstract

The current paper examines antecedents and consequences of perceiving conflict between gender and work identities in male-dominated professions. In a study among 657 employees working in 85 teams in the police force, we investigated the effect of being different from team members in terms of gender on employees’ perception that their team members see their gender identity as conflicting with their work identity. As expected in the police force as a male-dominated field, the results showed that gender-dissimilarity in the team was related to perceived gender-work identity conflict for women, and not for men. In turn, perceiving gender-work identity conflict was related to lower team identification for men and women. Although lowering team identification might enable employees to cope with conflicting social identities and hence protect the self, this may also have its costs, as lower team identification predicted higher turnover intentions, more burn-out symptoms, less extra role behavior, lower job satisfaction, lower work motivation, and lower perceived performance. Additionally, for women, experiencing support from their team members and team leader showed a trend to mitigate the relationship between gender-dissimilarity and perceived gender-work identity conflict, and a positive diversity climate was marginally related to less perceived gender-work identity conflict. The results show the importance of the team context in shaping a climate of (in)compatible identities for numerically underrepresented and historically undervalued social group members in order to hinder or protect their work outcomes.

Women (do not) belong here: Gender-work identity conflict among female police officers

“Although I generally feel good within our team, the feeling that as a woman you have to prove yourself even more to get the same appreciation as a police detective prevails.”

Female police officer participant

Nowadays, people often work in teams and teams shape people’s work experiences (Jackson, 1996), such as the extent to which they feel valued in their work team. Being a member of a social group that is a numerical minority can lead people to feel less valued in their work team or in the organization as a whole because of their social identity (Inzlicht and Good, 2006). The quote above from a female participant illustrates this can indeed be the case for women working in an organization such as the police force, which has a relatively short history of female employees and where women are still strongly underrepresented worldwide (Bureau of Justice Statistics, 2010; Statistics Belgium, 2010; Europol, 2013). She feels less valued in her team due to her gender and feels like she has to prove herself even more than her male colleagues to be equally valued. Put differently, she feels that her team members believe her gender to conflict with her work.

In the present research, we argue that the team context plays an important role in shaping employees’ perceptions of such gender-work identity conflict. We take an individual-within-the-team perspective on gender diversity and argue that differing from team members in terms of gender in this masculine environment affects women’s and not men’s perception of gender-work identity conflict. Additionally, we argue that employees who experience that their team members see their gender and work identities as conflicting can cope with this by attaching the self psychologically less to the team. Although reducing their team identification might enable employees to cope with conflicting identities and hence protect the self, this may have its costs for important work outcomes related to well-being, motivation, and performance at work (see, e.g., Ellemers et al., 1998; Ouwerkerk et al., 1999; Van Laar and Derks, 2003). Experiencing support from team members and from the team leader, and perceiving a positive diversity climate are examined as team contextual supportive factors that can buffer identity conflict for women.

## Gender-Dissimilarity in the Team and Gender-Work Identity Conflict

Work organizations and teams are increasingly diverse with also traditionally underrepresented groups finding their way into work fields in which they were less represented (Ely and Thomas, 2001). Research has primarily focused on effects of diversity at the group level, for example examining whether more diverse teams are more innovative and effective than less diverse teams (e.g., McLeod et al., 1996; Horwitz and Horwitz, 2007; Van Dijk et al., 2012). Other research has taken an individual-within-the-group perspective on diversity and how this affects individual outcomes (Tsui et al., 1992; Liao et al., 2004). This is important as individuals often struggle with being different from their team members, for example from gender-dissimilarity (Guillaume et al., 2012). Gender-dissimilarity is the difference between a focal group member and his or her group members with respect to gender. It could also be seen as a reflection of how prototypical a group member is within a group in terms of his or her gender (Oakes et al., 1998). In the current research we took such an individual-within-the-team perspective on gender diversity and examined how this related to employees’ meta-perception of how their team members view their gender and work identities (Frey and Tropp, 2006). We investigated whether being dissimilar from team members in terms of gender was related to employees perceiving more that their team members see their gender as conflicting with the work they do (i.e., perceived gender-work identity conflict).

We build on several theoretical frameworks for this research question. First, following the social identity approach (Tajfel and Turner, 1979; Turner et al., 1987), people categorize themselves and others into in- and out-groups based on observable similarities and differences (such as gender). Being dissimilar from others makes this category more salient (Wilder, 1984) and increases individuals’ tendency to expect that others will view them in terms of their group membership (Frey and Tropp, 2006). Thus, being dissimilar from other team members in terms of gender makes employees more aware of their gender and increases their expectation that they are viewed by other team members in terms of their gender. Adding to relational demography literature which is not conclusive on whether being dissimilar is more consequential for certain groups (see, e.g., Chattopadhyay et al., 2015), we argue that dissimilarity negatively impacts experiences in a team only when this dissimilarity is based on a social identity that is stigmatized within the given context. Indeed, the social identity that is made salient when one is dissimilar in a team is not neutral: being a woman in a traditionally male context tends to be associated with lower value to that identity (Branscombe and Ellemers, 1998; Inzlicht and Good, 2006; Van Laar et al., 2010). Women were not allowed into most European police forces until a few decades ago, and still are in a vast numerical minority. Also, in this setting masculine characteristics are strongly valued (Somvadee and Morash, 2008; Archbold et al., 2010). Attributes typically associated with being female are seen as not fitting or as incongruent with the attributes associated with being a police officer (Heilman, 1983; Eagly and Karau, 2002; Lyness and Heilman, 2006). That is, the prototypical police officer does not match the prototypical woman. This creates a perceived incongruity or lack of fit between being a woman and the work identity of a police officer. Related to this, negative stereotypes about women in the police force prevail (Somvadee and Morash, 2008; Archbold et al., 2010). Thus, being dissimilar from team members in terms of gender makes one’s gender salient and increases the expectation that other team members view you in terms of your gender. For women in the police force it also makes salient a social identity that is associated with negative stereotypes and lower value, and that is seen as incongruent with working in the police force. Therefore, we hypothesize that being gender-dissimilar from the team relates to a stronger perception that their team members see their gender as conflicting with their work identity for female police officers. Because of the masculine history and values of the organizational context, we expect that male officers will not have higher perceived gender-work identity conflict even when their gender differs more from that of their team members.

*Hypothesis 1*: Gender-dissimilarity is positively related to perceived gender-work identity conflict for female, but not male police officers.

## Coping with Gender-Work Identity Conflict and Work-Related Costs

People are not passive recipients of their environment when they experience that their social identity is devalued in a context, but they try to cope with it Van Laar et al. (2010). While employees might have relatively little influence on their team members’ perception of the conflict between their gender and work identities, they can cope with the conflicting identities by reducing their ties with the team. More specifically, perceived gender-work identity conflict is a cognitive concept that implies that the prototype of your gender group is not compatible with the prototype of your work identity (e.g., the prototypical police officer does not match the prototypical woman), hence leading to more marginal team membership (Oakes et al., 1998; Ellemers and Jetten, 2013). Level of team identification, on the other hand, refers to the affective ties of an individual to the team, which is more under an individual’s own control (Ellemers et al., 1999; Leach et al., 2008). Researchers have primarily examined group contexts in which more marginal (i.e., less valued) group members are motivated to become more prototypical and core group members (e.g., Levine and Moreland, 1994). However, this moving toward the group is not always the preferred trajectory (Ellemers and Jetten, 2013). In an environment in which the team communicates that you are a less valued team member as they perceive your gender and work identities as conflicting, attaching the self psychologically less to the group can be an important route to self-actualization, identity, and value (Ellemers and Jetten, 2013). Therefore, we hypothesize that employees will identify less strongly with their work team the more they perceive that their team members see their gender and work identities as conflicting. As outlined above, we expect women, but not men to experience more perceived gender-work identity conflict in face of gender-dissimilarity. However, to the extent that men do experience this conflict, we expect that they too will identify less with the team. In other words, we expected both male and female employees to endorse this coping strategy in the face of perceived gender-work identity conflict.

*Hypothesis 2*: Perceived gender-work identity conflict is negatively related to team identification.

While reducing identification with the team when perceiving more gender-work identity conflict among team members might enable employees to better cope with conflicting social identities, this has costs for their outcomes at work. Previous research has shown that members who identify less strongly with their group are less willing to contribute to collective goals (Ellemers et al., 1998; Tyler and Blader, 2000; Meeussen and Van Dijk, 2016), are less productive (Worchel et al., 1998; Meeussen et al., 2014a), and are less willing to put effort in the group beyond what is expected of them (Ouwerkerk et al., 1999; Van Knippenberg, 2000). We examined not only work motivation and performance at work, but also work-related well-being. While individuals can identify less with the team in response to gender-work identity conflict to protect the self (or more general well-being), this may come at the cost of less well-being *at work*. We hypothesize that lower team identification is linked to important work-related outcomes: lower work motivation, lower work satisfaction, lower perceived performance, less extra role behavior, more burn-out symptoms, and higher turnover intentions at work.

*Hypothesis 3*: Team identification is positively related to work motivation, work satisfaction, perceived performance, and extra role behavior, and negatively related to burn-out symptoms and turnover intentions.

Additionally, we examined the indirect relationship between perceived gender-work identity conflict and the work-related outcomes via lower team identification. We expect that a climate in which employees experience that their team members see their gender as conflicting with their work identity does not always directly translate into lower outcomes. Employees have different possibilities to respond in such a climate – for instance, increasing efforts at work to challenge the idea that their gender group may not fit with this work (Alter et al., 2010) or distancing the self from one’s gender group (Derks et al., 2011a). The current research examines lowering identification with the team as an important, but understudied, coping mechanism (Ellemers and Jetten, 2013). We expect that, to the extent that officers indeed do this, gender-work identity conflict will relate to lower work outcomes.

*Hypothesis 4*: Perceived gender-work identity conflict is indirectly negatively related to the work-related outcomes via lower team identification.

## Team Contextual Supportive Factors Buffering Gender-Work Identity Conflict

Lastly, we argue that supportive factors in the team context can reduce the negative impact of being gender-dissimilar from other team members for female police officers. First, experienced social support from team members and the team leader is argued to be important. Social support can include communication of emotional concern or comfort, affirmation, being able to turn to others for guidance and assistance when needed, and the provision of information (Wills, 1985; London et al., 2011; Richman et al., 2011). Such social support can reduce the impact of a stressor and the perceived stressfulness of an event or experience, hence offering a “stress-buffering” effect (Lazarus and Folkman, 1984; Cohen and Wills, 1985). Research on members of negatively stereotyped groups has shown that experiencing social support, or perceiving that it is available, is related to better achievement outcomes and engagement in work and education (Eccles, 1994; Walton and Cohen, 2007; Hartman and Hartman, 2008; Richman et al., 2011; Baysu et al., 2014). Another important team contextual supportive factor is a positive diversity climate. A positive diversity climate is the extent to which minority groups perceive the environment to be open toward their social group (Purdie-Vaughns et al., 2008; Gonzalez and Denisi, 2009; Plaut, 2010). Research has shown that perceiving a positive diversity climate is related to, amongst others, organizational commitment, organizational identification, feeling included, and lower turnover intentions for members of stigmatized groups (e.g., Gonzalez and Denisi, 2009; Meeussen et al., 2014b). Because of these key positive effects of experienced support and a perceived positive diversity climate for stigmatized group members’ outcomes in work and education, these team contextual supportive factors are examined as possible buffers for gender-work identity conflict for women in the police force context. We expect that the effect of gender-dissimilarity on perceptions of gender-work identity conflict for women is reduced when they experience support from their team members, when they experience support from their team leader, and when they perceive a positive diversity climate in their team. Given that we do not expect men to experience identity conflict when they are gender-dissimilar in their team (as their gender identity is not devalued within this context), we also do not expect contextual supportive factors to moderate this.

*Hypothesis 5*: Team contextual supportive factors (experienced support from team members, experienced support from the team leader, and perceived positive diversity climate) reduce the relationship between gender-dissimilarity and perceived gender-work identity conflict for female officers.

These hypotheses were tested in a large cross-sectional study among men and women working at the police force in a Western European country.

## Materials and Methods

### Participants and Procedure

The sample consisted of 789 employees of a police force in a Western European country.^1^ As the current study focuses on effects of gender-dissimilarity in teams and on team leader support, we excluded 132 participants who were team leaders themselves, resulting in a final sample of 657.^2^ The mean age of the participants was 43 years old (*SD* = 9.72, range: 21–64) and 60% were male (38% female, 2% unknown). For 54% of participants secondary education was their highest level of education, and 43% had a college or university degree (3% unknown). On average, participants had been working in the police force for 17 years (*SD* = 11.46), within their current department for 10 years (*SD* = 9.02), and in their current position for 9 years (*SD* = 8.12). Participants either had an executive position (55%) or a logistics or administrative position (42%; 3% unknown).

After the director of the organization had given consent for this research on “Diversity in the workplace,” team leaders of 122 teams distributed across all regions of the country were invited for participation. 15% (*n* = 18) of the team leaders could not be reached and 2% (*n* = 2) declined. Of the remaining 102 teams, 84% (*n* = 85) participated. The average size of a team was 12 people (*SD* = 6.81) and ranged from 3 to 33 people plus their leaders.^3^ Team leaders received and distributed the questionnaires among their members. Employees who consented to participate completed the survey individually during working hours, which took approximately 30 min. After completing the survey, employees returned their completed survey in a sealed envelope to their team leaders. Team leaders collected the envelopes from all team members and mailed them back to the researchers. The survey consisted of a wide range of questions on perceptions of diversity. The measures relevant for the current manuscript are detailed below. **Table 1** presents an overview of correlations between all variables.

**Table 1:** Correlations between all measures.

	1	2	3	4	5	6	7	8	9	10	11	12
(1) Gender												
(2) Gender-dissimilarity	0.40ˆ***											
(3) Team leader support	0.07	0.05										
(4) Team members’ support	0.07	0.09^∗^	0.55^∗∗∗^									
(5) Positive diversity climate	-0.05	0.07	0.40^∗∗∗^	0.47^∗∗∗^								
(6) Gender-work identity conflict	0.05	0.07	-0.08^∗^	-0.12^∗∗^	-0.10^∗^							
(7) Team identification	-0.02	0.01	0.49^∗∗∗^	0.52^∗∗∗^	0.46^∗∗∗^	-0.11^∗∗^						
(8) Turnover intentions	0.13ˆ**	0.09^∗^	-0.30^∗∗∗^	-0.16^∗∗∗^	-0.26^∗∗∗^	0.02	-0.35^∗∗∗^					
(9) Burnout symptoms	0.02	-0.04	-0.26^∗∗∗^	-0.18^∗∗∗^	-0.26^∗∗∗^	0.08^∗^	-0.27^∗∗∗^	0.33^∗∗∗^				
(10) Perceived performance	0.12ˆ**	0.04	0.24^∗∗∗^	0.17^∗∗∗^	0.16^∗∗∗^	-0.07	0.30^∗∗∗^	-0.16^∗∗∗^	-0.17^∗∗∗^			
(11) Extra role behavior	-0.08ˆ*	-0.02	0.18^∗∗∗^	0.12^∗∗^	0.15^∗∗∗^	-0.00	0.22^∗∗∗^	0.00	-0.02	0.32^∗∗∗^		
(12) Job satisfaction	0.01	-0.01	0.35^∗∗∗^	0.25^∗∗∗^	0.27^∗∗∗^	-0.03	0.50^∗∗∗^	-0.44^∗∗∗^	-0.38^∗∗∗^	0.40^∗∗∗^	0.25^∗∗∗^	
(13) Motivation	-0.00	0.02	0.36^∗∗∗^	0.26^∗∗∗^	0.30^∗∗∗^	-0.03	0.52^∗∗∗^	-0.40^∗∗∗^	-0.37^∗∗∗^	0.48^∗∗∗^	0.31^∗∗∗^	0.71^∗∗∗^

*^∗^*p* < 0.05; ^∗∗^*p* < 0.01; ^∗∗∗^*p* < 0.001; -1 = male, 1 = female.*

### Measures

Unless otherwise indicated, items were answered on a five-point Likert scale from (1) *strongly disagree* to (5) *strongly agree*. Measures are scored such that higher scores indicate stronger scores on the concept.

#### Gender-Dissimilarity

Gender-dissimilarity is the difference between a focal team member and his or her team members with respect to gender (Guillaume et al., 2012). Gender-dissimilarity was measured by calculating the Euclidean Distance between each respondent and his or her other team members (see for recommendations Harrison and Klein, 2007). For each individual team member the Euclidean distance was calculated by dividing the number of group members with a different gender by group size and then taking the square root of this fraction (Tsui et al., 1992; see also Jansen et al., 2016). For example, in a team with three men and two women, the Euclidean distance for the men equals √(2/5) = 0.63 and for women equals √(3/5) = 0.77. The Euclidean distance can range from 0 (all team members have the same gender as the focal team member) to nearly 1 (all team members have a different gender group than the focal team member). Women had a higher gender-dissimilarity compared to men, but the range and standard deviation were similar. For men, the mean Euclidean distance was 0.42 (*SD* = 0.24, range: 0.00–0.94); for women, 0.62 (*SD* = 0.21, range: 0.00–0.96).

#### Team Members’ Support

Experienced support from other team members was measured with “In my team, I can count on my colleagues when I experience difficulties at work” (*M* = 3.74, *SD* = 1.02; adapted from Van Veldhoven et al., 2002).

#### Team Leader Support

Experienced support from the team leader was measured with “In my team, I can count on my team leader when I experience difficulties at work” (*M* = 3.43, *SD* = 1.26) (adapted from Van Veldhoven et al., 2002).

#### Positive Diversity Climate

Positive diversity climate was operationalized in the current research as the perceived openness of the team to differences (items based on Luijters et al., 2008; Nakui et al., 2011). This was measured with eight items: e.g., “The ideas of colleagues who differ from each other complement each other” and “The differences between team members make this a valuable collaboration” (α = 0.70, *M* = 3.22, *SD* = 0.65).

#### Gender-Work Identity Conflict

The perception of gender-work identity conflict among team members was measured with “To what extent do you think that other colleagues consider your function as a police officer to be compatible with your gender?” [(1) *not at all* to (5) *very much*; *M* = 3.87, *SD* = 1.25]. The responses were reverse coded so that higher scores indicate more perceived gender-work identity conflict.

#### Team Identification

Participants’ identification with their team was measured with seven items (taken from Ellemers et al., 1999; Roccas et al., 2008). Example items are “I identify with the other members of my team” and “I feel strongly affiliated with this team” (α = 0.73, *M* = 3.51, *SD* = 0.66).

#### Work-Related Outcomes

Six measures assessed participants’ outcomes at work. First, *work motivation* was measured with the overall item “How motivated are you for your job?” [(1) *not at all* to (5) *very much; M* = 3.85, *SD* = 0.93]. Additionally, we measured *extra role behavior* with “I volunteer for things at the police force without being asked to” (*M* = 3.63, *SD* = 1.03, adapted from Van Veldhoven et al., 2002), and *turnover intentions* with two items: e.g., “I sometimes think of searching for work outside the police force” (*r* = 0.50, *M* = 2.26, *SD* = 1.20; adapted from Van Veldhoven et al., 2002). We measured *perceived performance* with two items: e.g., “How well did you execute the responsibilities of your job in the past month?” (*r* = 0.71, *M* = 4.18, *SD* = 0.67; based on Abramis, 1994). We measured employees’ job satisfaction with “Overall, how satisfied are you with your job?” [(1) *not at all* to (5) *very much; M* = 3.79, *SD* = 0.85] (based on the validated single-item measure developed by Dolbier et al., 2005; for a meta-analysis validating the use of single-item job satisfaction measures see also Wanous et al., 1997). Lastly, we measured employees’ burnout symptoms with three items (α = 0.83, *M* = 2.32, *SD* = 0.99): e.g., “I feel empty at the end of the day” (adapted from Van Veldhoven et al., 2002).

## Results

**FIGURE 1:**
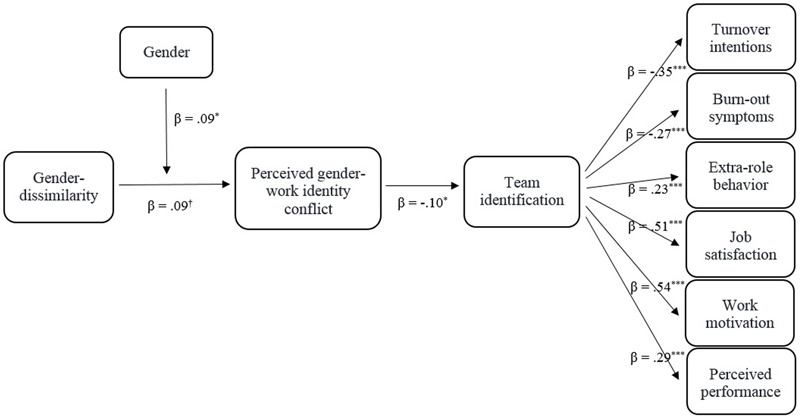
**Visual representation of the multilevel structural equation model (*CFI* = 0.96, *TLI* = 0.91, *RMSEA* = 0.06, *SRMR* = 0.03).** Gender-dissimilarity predicted only women’s perceived gender-work identity conflict. Gender-work identity conflict predicted team identification, which in turn predicted work outcomes for all participants. ^†^*p* < 0.10; ^∗^*p* < 0.05; ^∗∗∗^*p*< 0.001; -1 = male, 1 = female.

The data were analyzed using two-level multilevel analyses with a random intercept model in Mplus 5 (Muthén and Muthén, 1998). While all variables are individual-level measures, multilevel analyses allow us to control for the nested structure of data with employees nested in the 85 teams. We used maximum likelihood (ML) estimations, preferable when using 30 groups or more (Browne and Draper, 2000; Maas and Hox, 2004). Continuous independent variables were grand-mean centered. Standardized estimated effects are reported. We estimated a multilevel structural equation model to test the hypotheses that gender-dissimilarity predicts perceived gender-work identity conflict for female officers (Hypothesis 1), that perceived gender-work identity conflict predicts lower identification with the team (Hypothesis 2), that team identification predicts work-related outcomes (Hypothesis 3), and that perceived gender-work identity conflict is indirectly related to lower work-related outcomes via lower team identification (Hypothesis 4). This model is depicted in **Figure 1** and showed good model fit (*CFI* = 0.96, *TLI* = 0.91, *RMSEA* = 0.06, *SRMR* = 0.03, Bentler, 1990).

First, we looked into the relation between gender-dissimilarity and perceived gender-work identity conflict. Results showed a marginally significant main effect of gender-dissimilarity (β = 0.09, *p* = 0.06) on perceived gender-work identity conflict, such that employees who differed more from their team members in terms of gender perceived their team members to see more conflict between their gender and work identities. This marginal main effect was qualified by an interaction with gender: in line with Hypothesis 1, there was a significant interaction effect between gender and gender-dissimilarity on perceived gender-work identity conflict, β = 0.09, *p* = 0.03. For female police officers, gender-dissimilarity was related to higher perceived gender-work identity conflict (β = 0.17, *p* = 0.01), while for male police officers their gender-dissimilarity was unrelated to perceived gender-work identity conflict (β = -0.01, *p* = 0.83).^4^ There was no main effect for gender (β = 0.01, *p* = 0.89).

Confirming Hypothesis 2, the results showed that the more employees perceived that their team members see their gender and work identity as conflicting, the less they identified with their team, β = -0.10, *p* = 0.01.^5^ In turn, as expected in Hypothesis 3, team identification was significantly related to the six work-related outcomes: identifying with the work team related to lower turnover intentions (β = -0.35, *p* < 0.001), fewer burn-out symptoms (β = -0.27, *p* < 0.001), more extra role behavior (β = 0.23, *p* < 0.001), higher job satisfaction (β = 0.51, *p* < 0.001), higher work motivation (β = 0.54, *p* < 0.001), and higher perceived performance (β = 0.29, *p* < 0.001). Consistent with Hypothesis 4, perceived gender-work identity conflict was indirectly (and not directly, all *p*s > 0.16) related to work-related outcomes via team identification, and these effects were consistent and significant across the different outcome measures (turnover intentions: β = 0.04, *p* = 0.02; burn-out symptoms: β = 0.03, *p* = 0.02; extra role behavior: β = -0.02, *p* = 0.02; job satisfaction: β = -0.05, *p* = 0.01; work motivation: β = -0.05, *p* = 0.01; perceived performance: β = -0.03, *p* = 0.02).

Combined, the results show that being different from team members in terms of gender related to a stronger perception that their team members see their gender as conflicting with their work identity for female, but not male police officers. This perceived gender-work identity conflict was related to lower team identification, which in turn related to higher turnover intentions, more burn-out symptoms, less extra role behavior, lower job satisfaction, lower work motivation, and lower perceived performance.

Investigating Hypothesis 5 that team contextual supportive factors (experienced team members’ support, experienced team leader support, and perceived positive diversity climate) reduce the relationship between gender-dissimilarity and perceived gender-work identity conflict for female officers, we also added a three-way interaction between the team contextual supportive factors, participants’ gender, and gender-dissimilarity in the model. These interactions were not significant (Experienced team members’ support: β = -0.07, *p* = 0.12; Experienced team leader support: β = -0.07, *p* = 0.16; Positive diversity climate: β = -0.07, *p* = 0.17). Still, given that our results showed, as expected, an interaction between participants’ gender and gender-dissimilarity on perceived gender-work identity conflict, indicating only an effect for women, we performed tentative additional analyses looking into the role of contextual support factors for women only, as they are the ones who experience identity conflict when they are dissimilar (see **Table 2**).

**Table 2:** Standardized estimated effects (Maximum Likelihood) and standard errors on perceived gender-work identity conflict for female police officers.

β (*SE*)				
	***Buffer***		
	**Support team members**	**Support team leader**	**Positive diversity climate**
Intercept	1.72 (0.12)^∗∗∗^	1.72 (0.12)^∗∗∗^	1.67 (0.12)^∗∗∗^
Gender-dissimilarity	0.18 (0.06)^∗∗^	0.16 (0.06)^∗^	0.18 (0.06)^∗∗^
Buffer	-0.12 (0.07)^†^	-0.04 (0.08)	-0.14 (0.08)^†^
Gender-dissimilarity^∗^Buffer	-0.12 (0.07)^†^	-0.13 (0.08)^†^	-0.09 (0.08)

*^†^*p* < 0.10; ^∗^*p* < 0.05; ^∗∗^*p* < 0.01; ^∗∗∗^*p* < 0.001.*

**FIGURE 2:**
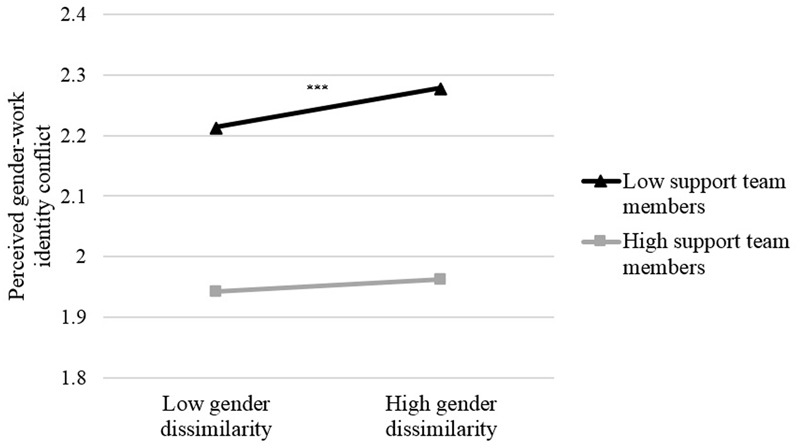
**Simple slopes indicating perceived gender-work identity conflict as a function of gender-dissimilarity and experienced support from team members for women (-1 SD or +1 SD above the mean).** ^∗∗∗^*p* < 0.001.

We first examined whether experienced support from team members could mitigate the negative effect of gender-dissimilarity on perceived gender-work identity conflict for women. There was a marginally significant negative relationship indicating that female police officers who experienced support from team members reported lower perceived gender-work identity conflict, β = -0.12, *p* = 0.090). This main effect was qualified by a marginally significant interaction between gender-dissimilarity and experienced team members’ support, β = -0.12, *p* = 0.082. An inspection of the simple slopes (see **Figure 2**) revealed that gender-dissimilarity was only related to higher perceived gender-work identity conflict when women experienced low support from their team members (*p* < 0.001), and not when experiencing high support from team members (*p* = 0.32). Thus, gender-dissimilarity was not related to perceived gender-work identity conflict anymore when experiencing high support from team members.

**FIGURE 3:**
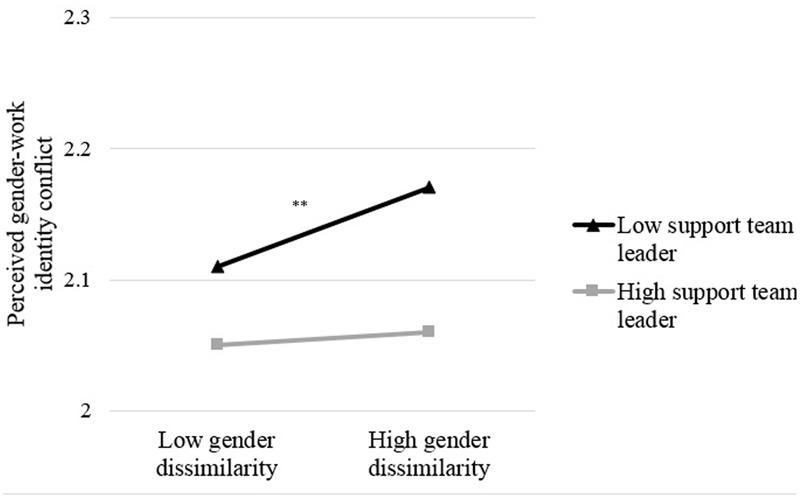
**Simple slopes indicating perceived gender-work identity conflict as a function of gender-dissimilarity and experienced support from the team leader for women (-1 SD or +1 SD above the mean).** ^∗∗^*p* < 0.01.

Second, there was no relationship between experienced team leader support and perceived gender-work identity conflict. However, there was a marginally significant interaction between gender-dissimilarity and experienced team leader support, β = -0.13, *p* = 0.095. Again, an inspection of the simple slopes (see **Figure 3**) revealed that gender-dissimilarity was related to higher gender-work identity conflict when women experienced low support from their team leader (*p* = 0.002), and that gender-dissimilarity was not related to perceived gender-work identity conflict anymore when experiencing high support from the team leader (*p* = 0.56).

There was no significant interaction between gender-work identity conflict and positive diversity climate. There was, however, a marginal negative relationship between positive diversity climate and perceived gender-work identity conflict indicating that female police officers perceived less gender-work identity conflict when perceiving a positive diversity climate in the team, independent of their gender-dissimilarity in the team, β = -0.14, *p* = 0.079.^6^

Thus, Hypothesis 5 could not be confirmed since there were no significant three-way interactions between participants’ gender, gender-dissimilarity and the team contextual supportive factors. Yet, our additional analyses do suggest that there may be potential in team contextual support factors for women, who are most vulnerable to identity conflict in teams where they are dissimilar. Results indicated that there was a trend for support from team members and team leaders to buffer perceived gender-work identity conflict when they are different from their team members in terms of gender.

## Discussion

The present study demonstrated the importance of dissimilarity from one’s work-team in shaping perceptions of conflicting identities for numerically underrepresented and historically undervalued social group members. This was shown among work teams in the police force in a Western European country – a setting where women are a numerical minority overall, where masculine characteristics are strongly valued, and where negative stereotypes about women prevail (Somvadee and Morash, 2008; Archbold et al., 2010). Additionally, we showed that perceiving conflicting identities in a team was related to lower identification with the team, which in turn related to lower important work-related outcomes.

### Gender-Dissimilarity Is Related to Gender-Work Identity Conflict for Female Police Officers

We demonstrated that for female police officers, being different from team members in terms of gender was related to perceiving more that their team members see their gender as conflicting with their work identity. The advantage of the current method in which we took an individual-within-the-team perspective on diversity is that we did not have to make a clear-cut distinction between majority and minority members, but could look for each individual how dissimilar they were compared to their team members (Jansen et al., 2016). So, although women are in a minority in the police force overall, and previous research has shown that compared to male officers female officers feel less valued in the police force (e.g., Archbold et al., 2010), the current research showed that this does not necessarily represent the experience of all women in the police force. Indeed, our data suggest that when they worked in more gender-balanced teams women perceived that their team members saw their gender as less conflicting with their work identity. Put differently, they did not feel less valued in their work team because of their gender.

For male police officers we did not find a relationship between gender-dissimilarity and perceived gender-work identity conflict. Previous research investigating gender differences in effects of gender-dissimilarity in teams or organizations on work outcomes has been inconclusive (Chattopadhyay et al., 2015), showing sometimes that effects may be stronger for women (e.g., Gonzalez and Denisi, 2009), and other times that gender-dissimilarity may actually be more consequential for men, who tend to be in the majority in work environments and are less used to gender-dissimilarity (e.g., Tsui et al., 1992), or that there is no difference in the effects for men and women (Jansen et al., 2016). Advancing relational demography literature, the current findings indicate that whether gender-dissimilarity negatively impacts experiences in a team depends not only on being dissimilar, but also on what is communicated about this social identity. That is, being dissimilar from other team members in terms of one’s social identity makes the social identity salient (Tajfel and Turner, 1979; Turner et al., 1987), and the value related to this salient identity then determines whether dissimilarity has negative effects: in traditionally male-dominated organizations like the police force (Somvadee and Morash, 2008; Archbold et al., 2010) dissimilarity makes an undervalued and negatively stereotyped social identity salient for women but not for men. The current research context provided an excellent opportunity to investigate this, as it allowed us to examine relationships in a masculine work environment in which women are both a numerical minority overall and in which negative stereotypes about women prevail (Somvadee and Morash, 2008; Archbold et al., 2010), but also one in which the range and variance in gender-dissimilarity was similar for male and female police officers. The reason this was the case was that employees with an administrative (vs. an executive) position were relatively more strongly represented in the current sample (42%) compared to the population of the police force (27%). Thus, our sample was not entirely representative for the police force overall, but this overrepresentation of administrative teams provided a more conservative test of our hypotheses where women are more represented and less negatively stereotyped. Future research could examine whether the current findings replicate in feminine contexts (e.g., nursing or teaching) where men are underrepresented. Do men in such contexts experience the same identity conflict in teams in which they are gender-dissimilar, and does it have similar consequences? In line with our interpretation of the findings in the current research, we would expect that this depends on whether the male identity is undervalued and negatively stereotyped in that organizational context.

### Coping with Gender-Work Identity Conflict and Work-Related Costs

To cope with experiences of conflict between identities, attaching the self psychologically less to the group can be used to protect self-identity and value (Ellemers and Jetten, 2013). In line with this, the present study found that employees identify less strongly with the team they work in when they perceive their team members to see their gender and work identities as more conflicting. Researchers have primarily examined group contexts in which less valued group members are motivated to become more prototypical and core group members (e.g., Levine and Moreland, 1994). However, moving toward the groups is not always the preferred trajectory (Ellemers and Jetten, 2013). Until recently, psychologists assumed that the need for belonging is better satisfied by being more typical in a group, as a core position is associated with greater acceptance and inclusion than a more marginal position (Baumeister and Leary, 1995). However, people are likely to hold multiple group memberships simultaneously that can satisfy their need for belonging (Ellemers and Jetten, 2013), and can hence identify less with certain groups they are part of to protect their self-identity and other social identities.

While this lower group identification might enable individuals to cope with conflicting social identities, this had its costs, since lower team identification related to lower work satisfaction, lower perceived performance at work, lower work motivation, less extra role behavior, higher turnover intentions, and more burn-out symptoms at work. This also implies that individuals may eventually start searching for work teams or occupations in which they feel more valued as a team member. This is consistent with a view of resilience in individuals to social identity threats. That is, individuals are quite able to cope with identity threat, finding ways to maintain general well-being, but this coping also has indirect and often hidden costs for the self or group (Derks et al., 2009, 2015; Van Laar et al., 2010; Ståhl et al., 2012), as evidenced also by the findings presented here.

In line with the view that people can be resilient when faced with identity threat, we found that perceived gender-work identity conflict was indirectly (via team identification), but not directly related to the work outcomes. Thus, only to the extent that individuals identify less with their team as a way of coping with identity conflict this is related to lower work-related outcomes. This shows that a climate in which employees experience that team members see conflict between one’s gender and work identities does not necessarily directly translate into lower outcomes, but that people can cope with this by lowering their identification with the team, which translates into lower work outcomes. In real life, individuals may use other strategies as well to cope with the experience of conflicting identities. For example, trying to convince the other team members that they should not consider their gender to be conflicting with their work in the team by emphasizing their masculinity or actively distancing themselves from other women (e.g., by emphasizing that they are different from other women at work, Derks et al., 2011a,b, 2016). In fact, Derks et al. (2011b) indeed found that women in the police force show this when reminded of their devalued status. So, individuals cannot only cope with perceived conflict between gender and work identities by distancing themselves from the work team to increase identity fit, but they can also distance from their gender group or try to resemble the other gender group more. Additionally, people could also reduce identification with their gender group. There are, however, reasons to believe that this is not very likely to happen. Gender is inherited and one of the most salient categories used to categorize oneself and others into in- and outgroups (particularly within organizational contexts, Hogg and Terry, 2000), and gender especially becomes salient when being gender-dissimilar from others (Tajfel and Turner, 1979; Turner et al., 1987). This makes it difficult to actually discard the identity when the team context makes this devalued social identity (in the current masculine context) salient, and when others repeatedly address one consistent with that identity (Branscombe and Ellemers, 1998). In line with this, recent research indeed found that underrepresented groups in outgroup domains identified more strongly with the outgroup and showed reduced concern for the ingroup, but did not identify less with the ingroup (Kulich et al., 2015). Investigating moderators influencing which coping mechanism is used when could be an interesting avenue for future research. For example, the current research focused on employees, showing that they can cope with conflicting identities by reducing their identification with the team. Previous research on self-group distancing as an identity management strategy, however, focused on employees with a leadership position (e.g., Derks et al., 2011a,b) and they might be less inclined to distance themselves from their achieved high status professional position. The status of one’s professional position could thus be an interesting moderator triggering different coping mechanisms. Additionally, future research could provide more insight in the differential (protective and harmful) effects of these different coping mechanisms on work outcomes. More generally, we hope that future research using longitudinal designs will be able to provide more insight in the dynamic interaction between the team context and negatively stereotyped group members’ coping with conflicting identities.

### The Role of Team Contextual Supportive Factors

The current findings indicate that conflict between gender and work identities has consequences for the person who experiences this identity conflict, as well as for teams and organizations they are working in. Therefore, we also examined whether such experiences of identity conflict when being in a gender-dissimilar team for female police officers could be mitigated by team contextual supportive factors. The results did not provide conclusive answers to this question. Although we did not find the expected interaction with participants’ gender, additional tentative analyses among female officers, who are most vulnerable to identity conflict in gender-dissimilar teams, did suggest that there may be potential in team contextual supportive factors. There was a trend for support from team members and team leaders to buffer identity conflict when they are different from their team members in terms of gender. This raises questions for future research. Perhaps being dissimilar from team members is not so easily overcome, particularly in the case of women in a highly male-dominated and masculine organization such as the police force. Team dynamics are embedded in the wider organizational structure and climate, which might constrain the impact of the supportive climate within the team. Thus, experiencing support and perceiving a positive diversity climate in the direct work environment might not be enough in such highly masculine organizations and are to be supplemented by more structural change in the broader work environment. Additionally, in future research it might be valuable to make a distinction between more emotional (e.g., communication of emotional concerns or comfort) and more instrumental (e.g., receiving guidance and assistance, provision of information) forms of social support (Wills, 1985). Certain types of social support might be better able to provide a buffer for experienced identity conflict than others.

### Limitations

A limitation of the current research is that our data was cross-sectional, and therefore we are not able to draw causal conclusions about the relations that were examined. For example, we examined perceived gender-work identity conflict in the team predicting individual team members’ identification with the team, but cannot conclude from the current study that perceiving conflicting identities causes lower team identification. Building on previous research, we know that perceiving that your team members see conflict between your gender and work identities indicates that one is a less valued team member because of one’s gender, and when people do not feel valued in a team, they may attach psychologically less to the team as an important route to self-actualization, identity, and value (Ellemers and Jetten, 2013). Because of this, it seems likely that perceiving conflict between identities leads to individuals themselves reducing their ties with the team. Yet, it is possible that on top of that, other group members may perceive more conflict between women and a work identity when they perceive women to have lower identification at work – hence the two may reinforce each other over time. Similarly, in the current research we expected that team identification would predict important work-related outcomes, which is in line with previous research that longitudinally and experimentally showed that team identification leads to better work-related outcomes (e.g., Worchel et al., 1998; Meeussen and Van Dijk, 2016). Still, it is possible that lower outcomes at work also lead to lower identification with the team. Follow up research could examine these relations in experimental or longitudinal designs to investigate causality.

## Conclusion

The current research showed the importance of the team context in shaping numerically underrepresented and historically undervalued social group members’ perception that their team members see their identities as conflicting. Findings indicated that as a woman being gender-dissimilar from team members in a masculine organizational context can trigger such perceptions of conflict between gender and work identities. This is important, because perceiving conflicting identities had negative implications for such individuals’ identification with the team, and consequently for their work outcomes. Understanding, how the team context can shape a climate of compatible identities for underrepresented group members in order to protect their work outcomes is an important question for future research. The current findings provided some indications that experiencing support from team members and team leaders might be able to shape such a climate.

## Ethics Statement

Before starting the questionnaire, participants agreed to an informed consent. They were informed that the researchers were interested in their experiences with, and perceptions of diversity in the police force. They were informed that participation was voluntary and could be discontinued at any moment during the study; and that their responses were anonymous and would be treated confidentially. Moreover, they were provided with room for questions and comments as well as contact information of the researchers. Additionally, they received a full debriefing at the end of the study.

Ethical approval was not required as per the local legislation at the time of the study commencement, and no ethics committee existed yet at the university for this type of research (only for clinical trials and medical research). However, this study was set-up in consultation with the police force, and the director of the organization approved the procedure of the study.

## Author Contributions

All authors contributed to the interpretation of the results and the writing of the manuscript. JV, LM, and CVL contributed to the development of hypotheses. LM and KP contributed to the development of the project. JV conducted the statistical analyses.

## Conflict of Interest Statement

The authors declare that the research was conducted in the absence of any commercial or financial relationships that could be construed as a potential conflict of interest.

## Footnotes

1 Due to agreements with the organization about anonymity, we do not state in which country this research was conducted.

2 We only included participants in the analyses of whom we could be sure that they did not have a leadership position in the team. Because of anonymity concerns, we could not ask the team leader to identify him/herself directly. We therefore identified the team leader using a bottom–up procedure consisting of several steps. Participants were asked whether they had a leadership position; if only one member of the team indicated they did, then this person was marked as the leader of the team. If two people indicated they did, then this mostly implied that one of them was the leader of the work team and the other had a leadership position higher up in the organization (which could be concluded by comparing the indicated number of people they led and its match with the number of participants from that team, and looking at the indicated number of levels and departments they led). They were both excluded from the current sample.

3 Team size estimates are based on the number of completed questionnaires in a team. This could be a slight underestimation for some teams, in case a team member was absent during the week of data collection.

4 The effect of gender-dissimilarity on perceived gender-work identity conflict for female officers remained when controlling for their position (executive vs. administrative), β = 0.23, *p* = 0.002. For male officers the relationship remained non-significant, β = -0.01, *p* = 0.87. Additionally, the interaction between gender-dissimilarity and gender also remained after controlling for participants’ position (executive vs. administrative), β = 0.12, *p* = 0.016.

5 Consistent with expectations, this relationship was not moderated by gender, β = -0.04, *p* = 0.64.

6 For male officers the relationship between gender-dissimilarity and perceived gender-work identity conflict did not depend on experienced team members’ support (β = 0.02, *p* = 0.68), experienced team leader support (β = 0.01, *p* = 0.86), and positive diversity climate (β = 0.05, *p* = 0.34). Additionally, for male police officers experienced team members’ support (β = -0.09, *p* = 0.12), experienced team leader support (β = -0.06, *p* = 0.26), and positive diversity climate (β = -0.03, *p* = 0.61) were not significantly related to perceived gender-work identity conflict.
